# A Rare Cause of Cyanosis Since Birth: Hb M-Iwate

**DOI:** 10.4274/tjh.galenos.2019.2019.0123

**Published:** 2019-11-18

**Authors:** Birgül Mutlu, Ebru Yılmaz Keskin, Ana Catarina Oliveira, Luis Relvas, Celeste Bento

**Affiliations:** 1 Doruk Yıldırım Hospital, Clinic of Neonatal Intensive Care, Bursa, Turkey; 2 Süleyman Demirel University Faculty of Medicine, Department of Pediatric Hematology and Oncology, Isparta, Turkey; 3 Centro Hospitalar e Universitário de Coimbra, Clinic of Hematology, Coimbra, Portugal

**Keywords:** Hb M-Iwate, Cyanosis, Methemoglobinemia

## To the Editor,

Cyanosis in an apparently healthy newborn baby may be caused by hemoglobin (Hb) variants associated with the formation of methemoglobin. Such Hb variants are collectively known as M Hbs [1]. Hb M-Iwate [alpha2 87(F8) His>Tyr, HBA2:c.262C>T] is one of the Hb variants associated with methemoglobinemia [2].

Many Hb variants have been reported so far from Turkey [3,4,5]. We report herein a newborn baby from Bursa, Turkey, with methemoglobinemia and (pseudo) cyanosis having Hb M-Iwate as the underlying cause. To our knowledge, this is only the second report of Hb M-Iwate from Turkey, and more than four decades have passed since its first observation in Turkey in a 21-year-old male by Ozsoylu [6]. In addition, our case represents the first case of Hb M-Iwate from Turkey identified through genetic analysis of the α-globin chain gene (*HBA*).

The boy, born at term to a 32-year-old mother, was noted to be cyanotic immediately after birth. He had findings of dyspnea and he received oxygen by hood.

In the family history, the mother had history of cyanosis, particularly in the peroral area, and was otherwise healthy. In addition, the maternal grandfather and his mother, who had migrated from Thessaloniki (Greece), also had a history of cyanosis.

The oxygen saturation (SpO_2_) of the baby, measured by pulse oximeter, was between 50% and 60%. Administration of oxygen did not result in an increase of the measured SpO_2_. In venous blood gas analysis, pH was 7.43, pCO_2_ was 34.6 mmHg, pO_2_ was 45.3 mmHg, and the p_50_ value was 39.2 mmHg (normal range: 22.6-29.4 mmHg). Methemoglobin relative concentration was 13.5% (normal: <1.5%). Complete blood count testing (Table 1) and echocardiographic examination were both normal.

In the follow-up of the case, findings of dyspnea resolved by the 3^rd ^postnatal day, although cyanosis persisted. The baby was discharged on the 4^th^ day in good condition.

**Figure 1:**
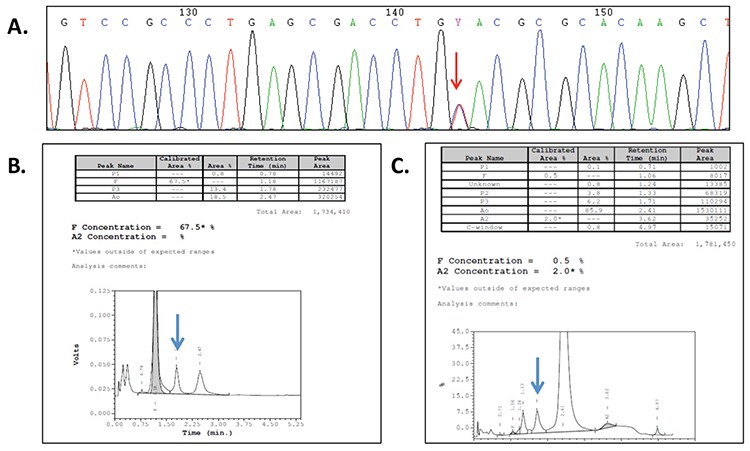
A) DNA sequence of a segment of exon 2 of the *HBA2* gene showing the c.262C>T mutation. B) HPLC of the propositus (newborn). Peaks corresponding to Hb F (67.5%), Hb M-Iwate (identifield as P3) (13.4%; arrow), and Hb A (18.5%) are observed. C) HPCL of the mother. Peaks corresponding to Hb F (2.5%), Hb M-Iwate (identified as P3) (6.2%; arrow), Hb A2 (2.0%), and a small fraction (C-window), corresponding to HbA2var (a^Iwate^_2_^δ^_2_), are observed.

Genetic analysis by Sanger sequencing of the *HBA* genes identified a pathogenic variant, HBA2:c.262C>T, corresponding to the already described Hb M-Iwate [alpha2 87(F8) His>Tyr] in the propositus and in his similarly affected mother (Figure 1A). This Hb variant could be detected by high-performance liquid chromatography (HPLC) (Beta-Thalassemia Program, Bio-Rad) (Figures 1B and 1C).

The M Hbs are transmitted in an autosomal dominant fashion and the existence of familial cyanosis with this pattern of inheritance was first recognized in Japan more than 200 years ago. In the 1950s, Shibata et al. [7] discovered the cyanosis to be due to an abnormal Hb in a large family with about 70 affected individuals. This abnormal Hb was later given the name Hb M-Iwate. In the vivid description of the clinical picture by Shibata et al. [8], “The patients with this disease are cyanotic from childhood, looking like a man who has been swimming in a cold water pool for a long time”.

In conclusion, M Hbs should be considered in the differential diagnosis of cyanosis in the newborn period. HPLC can identify the presence of an Hb variant but gene sequencing is necessary for the identification of abnormal variants. Except for cosmetic consequences, the clinical course of patients with Hb M-Iwate is unremarkable.

## Figures and Tables

**Table 1:**
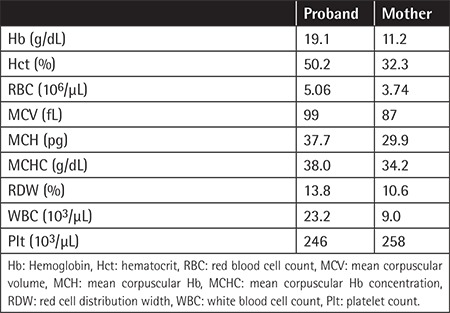
Complete blood count parameters of the proband and his mother on the first postnatal day.
